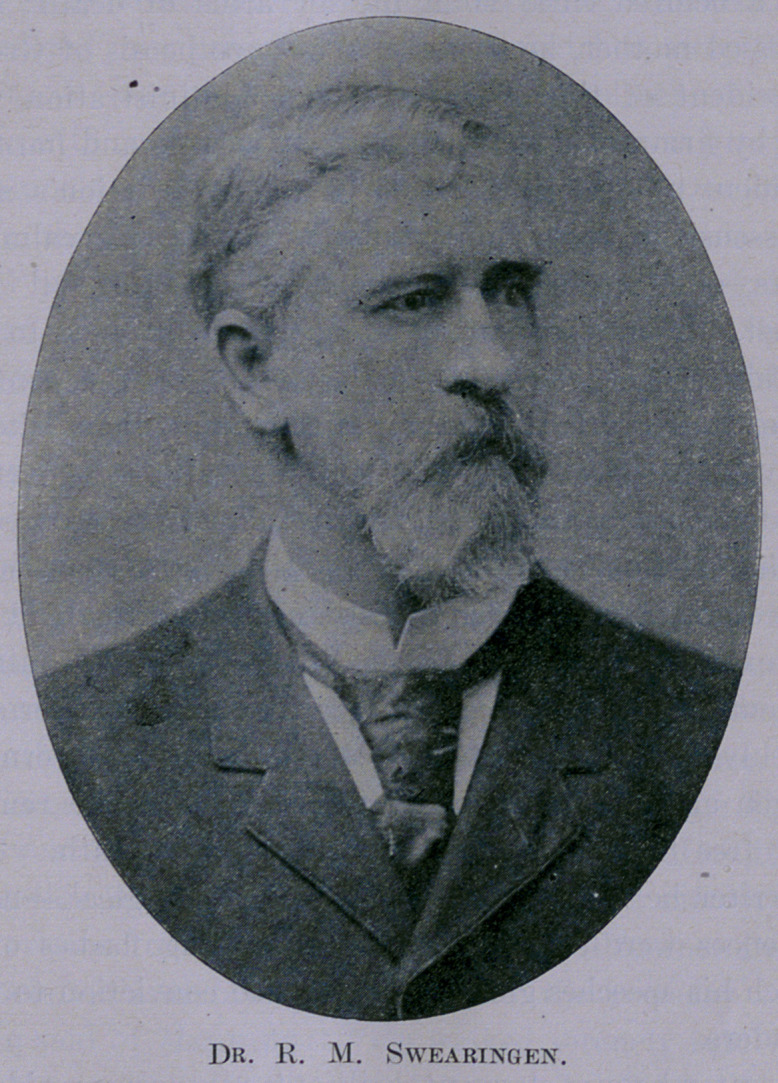# Memorial Address*Delivered in the Auditorium at Waco, Texas, on the occasion of the twenty-third annual meeting of the Texas State Medical Association, April 26, 1900.I have had so many calls for copies of this address, and the edition being long since exhausted, that I am induced to republish it. And I take advantage of our annual reunion to do so as being especially fitting. There are hundreds of members and hundreds of subscribers to the *Texas Medical Journal* who have never seen it and who, I know, will be glad to get a copy as a souvenir of the illustrious, loved and lost Swearingen, who was dear to the heart of every physician and every man, woman and child in Texas.F. E. Daniel.

**Published:** 1906-04

**Authors:** F. E. Daniel

**Affiliations:** Austin, Texas


### TEXAS MEDICAL JOURNAL.

ESTABLISHED JULY, 1885.

PUBLISHED MONTHLY.—SUBSCRIPTION $1.00 A YEAR.

VOL. XXI. AUSTIN, APRIL, 1906. No. 10.

The publisher is not responsible for the views of contributors.

### Memorial Address.*

BY F. E. DANIEL, M. D., AUSTIN, TEXAS,

IN MEMORY OF SWEARINGEN.

*Mr. President, Fellows of the Texas State Medical Association, Ladies and Gentlemen:*

By the courtesy of an invitation embodied in a resolution adopted at the last meeting of this Association, it is my privilege, as it is an honor, to pay tribute to the memory of one of our brightest and best, whose soul has been translated to the realms of eternal rest:—Dr. Richard Montgomery Swearingen.

The State Medical Association,—the people of Texas,—the medical profession of America,—need no eulogy upon his life, character and services,—his talents,—his successes in that sphere of life to which he was called. They need not be told of his virtues in private life. No recapitulation of his soldierly deeds in defense of the South in her unequal struggle for independence, or his services to Texas as her chief sanitary officer; no allusion to his wide reputa-

*Delivered in the Auditorium at Waco, Texas, on the occasion of the twenty-third annual meeting of the Texas State Medical Association, April 26, 1900.

I have had so many calls for copies of this address, and the edition being long since exhausted, that I am induced to republish it. And I take advantage of our annual reunion to do so as being especially fitting. There are hundreds of members and hundreds of subscribers to the *Texas Medical Journal* who have never seen it and who, I know, will be glad to get a copy as a souvenir of the illustrious, loved and lost Swearingen, who was dear to the heart of every physician and every man, woman and child in Texas.

F. E. DANIEL.

tion as a skilled physician and learned sanitarian is necessary. It is all familiar to them, and he will live in their memory, and the influence of his example will be felt, as a star, blotted from the firmament, will still pour its light upon us.

But that those who come after us,—some to guide the destinies of this great State,— others to guard its portals with a sanitary cordon; some to preside over, —others to sit in the councils of this Association may learn of his beautiful life, his rare qualities of head and heart, and know the warm place he occupied in the affections of the present generation — the measure of usefulness he filled in his busy life, it is meet that we attempt to formulate it into words for record in the archives of the State Association of physicians, that, inspired with his illustrious example, our successors may labor to emulate it.

I knew Dr. Swearingen well. In his public and his private life, —in the intimacy of his happy home,—associated with him officially many years, my opportunities for properly estimating his worth were exceptional. I have seen him under almost every conceivable circumstance, and I can sincerely say that never in my life have I known so perfectly rounded out and complete a manhood. In every relation of life he was simply grand. He exemplified the loftiest ideal of splendid manhood. Yet he was as gentle as a child, and as free from guile. His generous heart was a stranger to anything approaching selfishness, and no unworthy motive or sordid interest ever actuated him. He was the calm, dignified, courteous, courageous man,— the peerless gentleman, whose knightly crest was never lowered in the presence of any man, any danger or trial, or under any circumstances. He had the heart of a lion,—but it was actuated by the sentiment and emotion of a gentle woman. Its every throb was for humanity. I have seen him approached by the high,—the powerful,—the lowly. His manner was the same to all—gentle, considerate, patient. The humblest of God's creatures could enter his presence with the assurance of kindly sympathy, and the needy never departed empty-handed. Considerate of the rights and opinions of others, he ever put the best construction upon every act of which it was susceptible.

Yet, measured by the world's estimate of greatness he was not a great man. He led no victorious hosts to inglorious conquest; he waded thro' no seas of slaughter to a throne. He ruled over no great State or nation. He negotiated no great treaties by which alien people were linked in fraternal bonds, nor speeded the white wings of commerce around the globe. He wrote no great books; made no great discoveries in the field of science, nor achieved distinction in any of the arts of peace or war. That is the world's idea of greatness; those men only who do "great" things live in history. But greatness, like distance, space and time, is not measured by any fixed standard; it is not absolute, but relative. If to live right and do right; if to walk upright in the eyes of God and man,—to be pure in heart, just, and gentle, brave and generous and useful; to love humanity; to do good for righteousness' sake;

DR. R. M. SWEARINGEN.

to do one's whole duty as he sees it fearlessly and fully is to be great, Swearingen was a great man. He was endowed with many of the elements of greatness, and under a different environment might have attained to eminence as an advocate, a statemsan, legislator, diplomat or author. In his limited sphere of action he utilized to the fullest his God-given "talent," nor let it rust. With him the practice of medicine was largely a mission of mercy. He refused his services to none; compelled none to pay him. He ever persuaded the erring, uplifted the weary and comforted the distressed,—emulating the Great Physician, who proclaimed "Peace on earth: Good will toward Man." He was a successful physician, whose very presence carried hope and healing to the afflicted, equally in the home of prosperity and in the hut of poverty. By the bedside, perhaps, of some mite of humanity whose little torch was fast flickering and fading,—watching in the still hours of night, when the world's great eye is shut, its ear closed and the pulse of traffic is still, he fanned the little spark to life again, and snatched a beloved child from the icy arms of death; there,—to the distressed mother, he shone the very apotheosis of Greatness.

As president of this Association his administration was characterized by firmness and gentleness; by success and harmony. He was president at a critical period of the Association's usefulness. When dissensions arose, his temperate words, his calm, rational arguments,—his persuasive eloquence were like "oil upon the waters cast." Where discord entered, peace remained to rule.

As Health Officer he was popular, and had the confidence of the whole State. He held that position more than fifteen years, consecutively, except during Ross' administration as governor. He remodeled the quarantine laws and formulated and secured the passage of the existing act. In the execution of this law he was signally successful; for no foreign pestilence found foothold in Texas, and the extensive epidemic of smallpox which had gained headway in some forty localities during Governor Ross' term of office was quickly suppressed when, reappointed by Governor Hogg, Swearingen again assumed management. The State remained remarkably free from smallpox to the date of his death.

As a writer he was forceful, clear, concise; logical,—not ornate. His sentences were stripped of those dazzling flashes of rhetoric with which his speeches glowed, and carried conviction to the minds of all readers.

As an orator he was eloquent, brilliant, persuasive; widely known as one of our most gifted extempore speakers. He had a sublime faith in an all-wise and beneficent Creator, and clearly portrayed it, and revealed his hope of immortality in glowing eloquence in his chaste and scholarly address to the literary societies of the Texas University, the subject being, "The Conservation and Correlation of Forces as a Basis of Belief in Immortality." It was a masterpiece of oratory, and sparkled with gems of rhetoric. At times the thoughts touched the sublime, and were flashed forth in language of living light. Deeply versed in the resources of the English language, he was master of the art of diction, and swayed the hearts and swept along the thoughts of all hearers.

He was widely known as a yellow fever expert; and after the great epidemic of 1878 was appointed on a congressional committee to investigate and report upon its origin, mode of development, etc. The result of the report made by this commission was the creation by Congress of a National Board of Health, now merged into the Marine Hospital Bureau.

When pestilence stalked the land; when the "saphron demon" blew his blighting breath over the fair Southland, and the destroying angel hovered over peaceful homes and snatched away the fairest and best,—it was then that this gallant soldier of sanitary science shone with conspicuous splendor. There he was great. Like the white plumes of Navarre, always riding above the storm of battle where the strife was deadliest, the crest of this modern knight could be seen as he battled with the hosts of darkness and death. When his native State, Mississippi, was in the throes of the great yellow fever epidemic of 1878, and the peaceful and happy little town of Holly Springs, under the impulse of generous sympathy and that hospitality ever characteristic of the South, threw open her doors to the terror-stricken refugees from the beleagured towns, and became, herself, the storm-center of the pestilence, Swearingen and Manning,—the brave, the gallant, the lovable Manning,—were prompt to go to her relief. Manning fell. Many noble volunteer physicians and nurses fell, laying down their lives cheerfully in the cause of humanity,—in obedience to that power that impels a heroic soul to self-immolation in the discharge of duty. Swearingen held the hand of the dying Manning, and later, before this Association, paid a tribute to his memory in an oration of love-inspired eloquence which today illumines the pages of the Association's archives, and lives in the memory of those present. He said of Manning: "He walked serenely into the valley of death to surpass all others in doing good. * * * It was called 'rash,' because he had never had yellow fever. * * * If his going to beautiful flower-crowned Holly Springs to battle and die with her brave men and women was a rash act,—in all reverence I say it was rash in the Savior we adore to leave the courts of Heaven to redeem a fallen race. The one came at the bidding of the Father; the other went, impelled by the same spirit that gave splendor to the Hill of Calvary, when temples and stars and heaven and earth reeled and rocked to the martyrdom of a God."

A touching memento of that dread visitation exists today. It is an epitaph written by Swearingen upon the white-washed walls of a room in the court house in which a Sister of Mercy died, the last but one, of the band who perished there. While all else of the walls is defaced, this lead pencil inscription has been respected and left intact. It illustrates at once Swearingen's humanity, his sympathetic nature and his beautiful poetic sentiment. He wrote:

"Within this room, September, 1878, Sister Corintha sank into the sleep eternal. Among the first to enter this realm of death, she was the last, save one, to leave. The writer of this humble notice saw her in health, gentle but strong, as she moved with noiseless step and serene smile through the crowded ward. He saw her when the yellow-plumed angel threw his golden shadow over the last sad scene, and eyes unused to weeping paid the tribute of tears to the brave and beautiful 'Spirit of Mercy.'

"She needs no slab of Parian marble,
With its white and ghastly head,
To tell the wanderers in the valley
The virtues of the dead.
Let the lily be her tombstone,
And the dew-drops pure and bright
The epitaphs the angels write
In the stillness of the night."

☘ ☘ ☘

The present is not the time nor the occasion to speak of Dr. Swearingen's splendid record as a soldier in the service of the South; that is history, and presents an unbroken story of dauntless courage and deeds of daring. Yet it may not be amiss to say, that at the age of twenty-four, he was at the head of a splendid troop of cavalry,—always at the front,—and passed through scenes and events that rival the most thrilling episodes of feudal times. In the privacy of our intercourse, with the modesty characteristic of the man, he would tell me some of his experiences, giving credit, always, to his "men." There was one event, however, of which he could make no other than himself the hero. Left sick at the house of a Tennessee gentleman in the territory into which his troops had made a raid, he was soon in the enemy's lines. Nursed back to health by the pretty daughter of the house, he fell a victim to her beauty and graces. They were married, and he rejoined his command. He ventured back alone,—at night—to visit his bride; the house was surrounded and he was captured,—not by the enemy proper, —but by a gang of those lawless men known as "guerillas." He was bound, gagged, and thrown in a fence corner to await his doom. At daylight he was led out to be shot. One of the band whom the bride's father had befriended in some emergency had sufficient influence to secure his release, and he was put thro' the lines. Upon such slim threads sometimes hang important events. The unknown young cavalryman became the illustrious physician and sanitarian, admired for his virtues and abilities, esteemed for his worth, and famous for his services, alike in war, and in the less tempestuous walks of life.

In the field of romance, fact or fiction, we scarcely find a parallel. The reckless daring, the romance of the times and environment; the union of these two heroic young souls, in the mountain fastnesses of a rugged country, the home of outlawry and the den of guerillas, a section of country swept by the storm of war, whose waves ebbed and flowed across it, back and forth with the advance or retreat of either army,—their separation, and final reunion in our own country,—their subsequent trials and hardships, and final triumphs in attaining wealth and distinction, and an evening of life crowned with happiness and serene content, furnish a theme for the Troubadour, worthy of the days when knighthood was in flower,—unexcelled in song or story.

☙ ☙ ☙

When all-conquering death came to the bosom of his home he met his end with that calm courage and unruffled front with which he had met all trials in life. It was peaceful,—beautiful. "Sustained and soothed by an unfaltering trust," he "approached his grave as one who wraps the drapery of his couch about him and lies down to peaceful dreams." The last thought that crossed his mind was one of "good will toward man." His last act, when the tongue refused its office, was to laboriously write, with a lead pencil, these words: *"I go out upon the great Unknown, without an unkind thought or feeling toward any living creature."*

Surely, such men were created for a purpose and not to perish. Such souls never die. In the grand and incomprehensible scheme of the limitless universe,—instinct with Deity,—vibrant with Life and Love in every molecule of the ethereal realms of the upper deep;—away,—beyond the remotest world revealed to man by his ingenuity; beyond the farthest star, whose light, outstripping the lightning's flash in incredible speed requires centuries to reach our planet, there is an abode of eternal peace and rest. There dwells the Great First Cause, the Source and Center of that all-pervading Energy we call "Life,"— whose manifestations in myriad forms make up all the pleasing, grand and beautiful phenomena of human environment; an Intelligence, recognized by man, but whose Purpose is inscrutable. Every organism, whether animal or vegetable, is but a specialized form from a primitive cell, and, physically, is but an aggregation of cells. In the germ of every cell there is, indwelling, the vital principle,—life itself. These cells are not only "living," but are endowed with a potentiality surpassing the ken of man,—to reproduce themselves indefinitely; and with an intelligence which guides and directs them in the upbuilding of the organism; they know where to go and what to do, and do it, unerringly and well. In the acorn slumbers the giant oak; in the tiniest seed, a grand flower-plant, which, germinating within its shell, bursts its bonds, and building up its stalk and stems, unfolds its leaves to kiss the sunlight and drink the dews of heaven. Under the guidance of that mysterious intelligence, in Nature's laboratory the inorganic elements, drawn from the earth, are wrought into living tissue, and its life work is completed and crowned with a burst of bloom, whose dazzling tints please the eye, —whose perfume, wafted on the breezes, fills the air with fragrance. Thus it is with man. The soul-germ is implanted by God in the tiniest embryonic cell,—an atom of protoplasm. It grows within the heart and expands with every good deed,—till at what we call "death" it bursts its earthly bindings, soars unto the Eternal, and blossoms in Immortality!

Gentle, brave, true Swearingen! Loyal friend,—spotless man, —Farewell! We meet; we miss thee. And while we mourn thine absence from our midst, we are consoled and made strong by the faith that in the realms of Celestial Light, thy great soul will forever shine, a Star of Day.

## Figures and Tables

**Figure f1:**